# Supplementary material for: Tumor-intrinsic YTHDF1 drives immune evasion and resistance to immune checkpoint inhibitors via promoting MHC-I degradation
Source: Nat Commun. 2023 Jan 17;14:265. doi: 10.1038/s41467-022-35710-7 (PMC9845301; doi:10.1038/s41467-022-35710-7)
Supplement: Supplementary file 1 — Supplementary Information [file 41467_2022_35710_MOESM1_ESM.pdf]

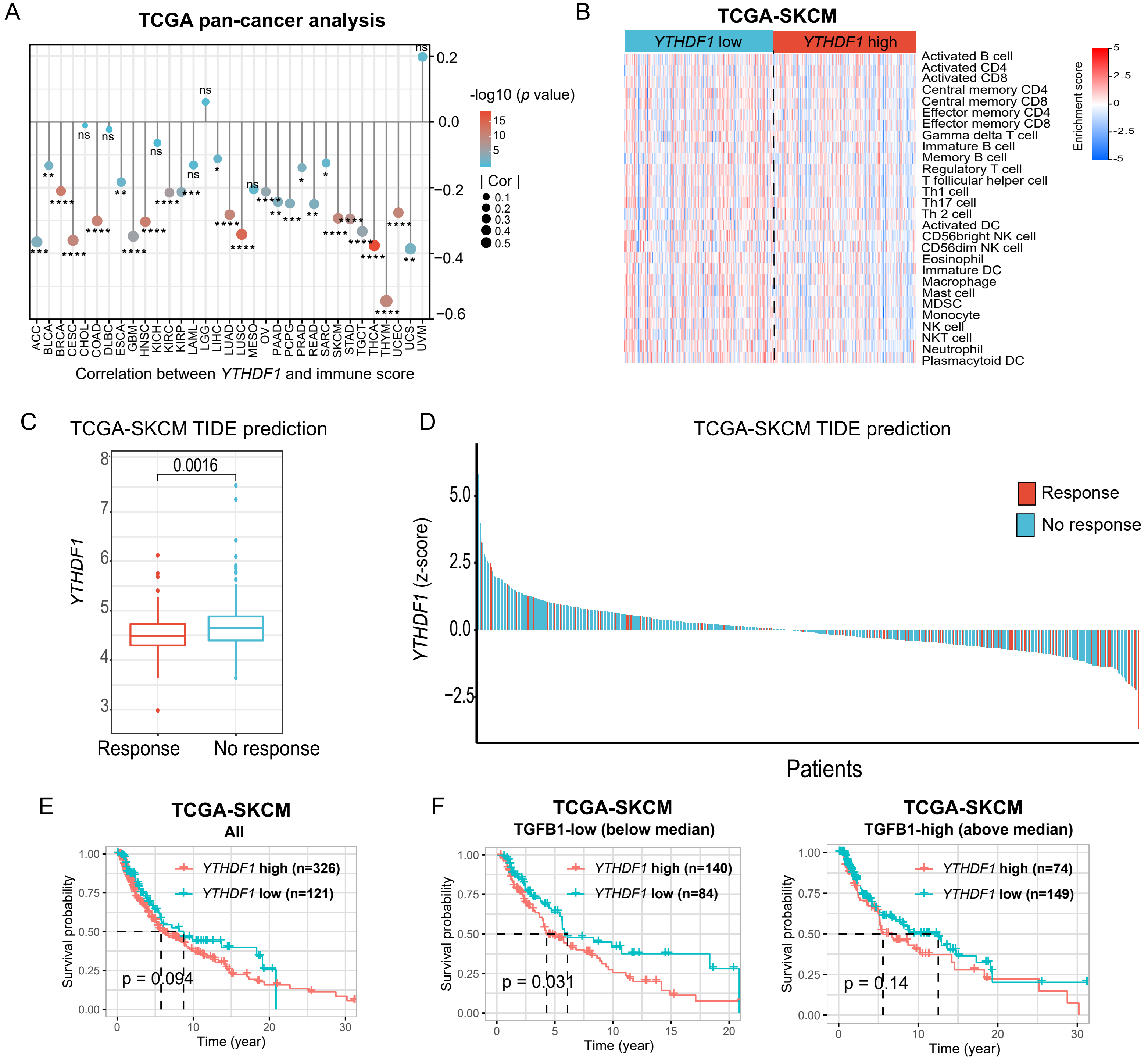

A
TCGA pan-cancer analysis
-log10 (p value)
15
10
5
| Cor |
0.1
0.2
0.3
0.4
0.5
0.2
0.0
−0.2
−0.4
−0.6
ACC BLCA BRCA CESC CHOL COAD DLBC ESCA GBM HNSC KICH KIRC KIRP LAML LGG LIHC LUAD LUSC MESO OV PAAD PCPG PRAD READ SARC SKCM STAD TGCT THCA THYM UCEC UCS UVM
Correlation between YTHDF1 and immune score
B
TCGA-SKCM
YTHDF1 low
YTHDF1 high
Activated B cell
Activated CD4
Activated CD8
Central memory CD4
Central memory CD8
Effector memory CD4
Effector memory CD8
Gamma delta T cell
Immature B cell
Memory B cell
Regulatory T cell
T follicular helper cell
Th1 cell
Th17 cell
Th 2 cell
Activated DC
CD56bright NK cell
CD56dim NK cell
Eosinophil
Immature DC
Macrophage
Mast cell
MDSC
Monocyte
NK cell
NKT cell
Neutrophil
Plasmacytoid DC
Enrichment score
5
2.5
0
-2.5
-5
C
TCGA-SKCM TIDE prediction
0.0016
YTHDF1
Response
No response
D
TCGA-SKCM TIDE prediction
Response
No response
YTHDF1 (z-score)
5.0
2.5
0.0
-2.5
Patients
E
TCGA-SKCM
All
YTHDF1 high (n=326)
YTHDF1 low (n=121)
p = 0.094
Survival probability
Time (year)
F
TCGA-SKCM
TGFB1-low (below median)
YTHDF1 high (n=140)
YTHDF1 low (n=84)
p = 0.031
TCGA-SKCM
TGFB1-high (above median)
YTHDF1 high (n=74)
YTHDF1 low (n=149)
p = 0.14
Survival probability
Time (year)

Supplementary Figure 1. The association of YTHDF1 expression with immune cell infiltration and the response to ICI therapy. (A) Spearman correlation of *YTHDF1* expression with the immune score. RNA-seq data from a total of 10363 individuals in the TCGA, representing 33 different types of tumors, were examined. **(B)** Heatmap presenting immune cell infiltration in the *YTHDF1*-high and *YTHDF1*-low groups. The standard used to define high and low *YTHDF*1 expression was determined based on median *YTHDF1* expression. ssGSEA was performed to estimate the infiltration of 28 immune cell types according to the summarized immune gene signature. **(C)** Box plot showing the differential expression of *YTHDF1* in the responder (n = 100 biologically independent samples) and nonresponder (n = 370 biologically independent samples) groups using RNA-sequencing data from the TCGA-SKCM dataset. Tumor immune dysfunction and exclusion (TIDE) analysis was conducted to predict the ICI response. TIDE (http://tide.dfci.harvard.edu/) is an analytic technique that enables the prediction of the ICI response. The upper, middle and lower horizontal lines of the box represent the upper, median and lower quartile respectively. Whiskers depict the smallest or largest values within 1.5-fold of the interquartile range, and the points outside the box represent outliers. Two-tailed Wilcoxon test. **(D)** A waterfall plot showing *YTHDF1* expression (z-score) for responders and nonresponders in the TCGA-SKCM dataset. **(E, F)** Prognostic value of *YTHDF1* in the TCGA-SKCM dataset (E) and its subgroup analysis in *TGFB*-high and *TGFB*-low groups (F). Two-tailed log-rank (Mantel-Cox) test. Source data are provided as a Source Data file.

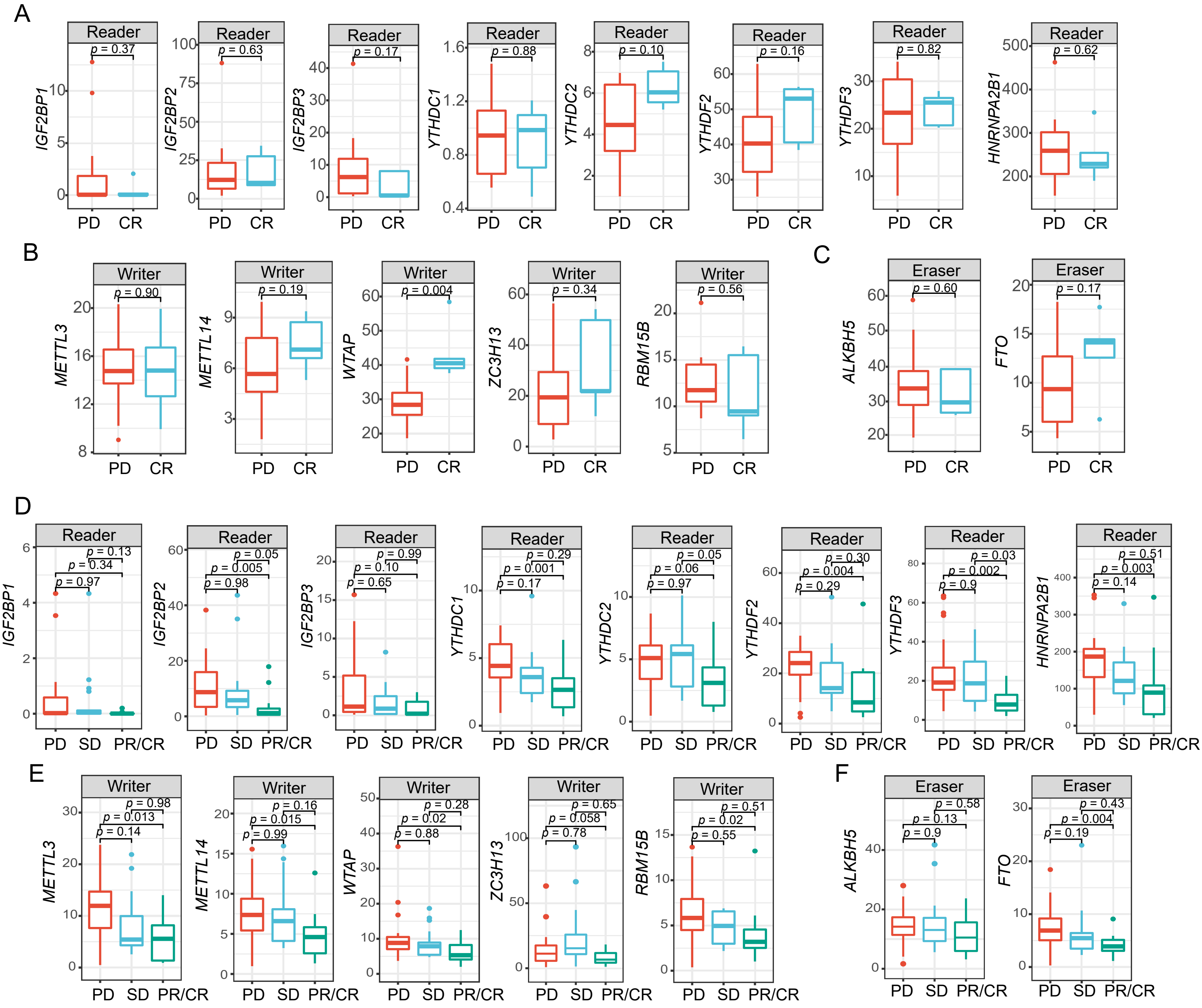

A
Reader
IGF2BP1
p = 0.37
PD CR
Reader
IGF2BP2
p = 0.63
PD CR
Reader
IGF2BP3
p = 0.17
PD CR
Reader
YTHDC1
p = 0.88
PD CR
Reader
YTHDC2
p = 0.10
PD CR
Reader
YTHDF2
p = 0.16
PD CR
Reader
YTHDF3
p = 0.82
PD CR
Reader
HNRNPA2B1
p = 0.62
PD CR
B
Writer
METTL3
p = 0.90
PD CR
Writer
METTL14
p = 0.19
PD CR
Writer
WTAP
p = 0.004
PD CR
Writer
ZC3H13
p = 0.34
PD CR
Writer
RBM15B
p = 0.56
PD CR
C
Eraser
ALKBH5
p = 0.60
PD CR
Eraser
FTO
p = 0.17
PD CR
D
Reader
IGF2BP1
p = 0.13
p = 0.34
p = 0.97
PD SD PR/CR
Reader
IGF2BP2
p = 0.05
p = 0.005
p = 0.98
PD SD PR/CR
Reader
IGF2BP3
p = 0.99
p = 0.10
p = 0.65
PD SD PR/CR
Reader
YTHDC1
p = 0.29
p = 0.001
p = 0.17
PD SD PR/CR
Reader
YTHDC2
p = 0.05
p = 0.06
p = 0.97
PD SD PR/CR
Reader
YTHDF2
p = 0.30
p = 0.004
p = 0.29
PD SD PR/CR
Reader
YTHDF3
p = 0.03
p = 0.002
p = 0.9
PD SD PR/CR
Reader
HNRNPA2B1
p = 0.51
p = 0.003
p = 0.14
PD SD PR/CR
E
Writer
METTL3
p = 0.98
p = 0.013
p = 0.14
PD SD PR/CR
Writer
METTL14
p = 0.16
p = 0.015
p = 0.99
PD SD PR/CR
Writer
WTAP
p = 0.28
p = 0.02
p = 0.88
PD SD PR/CR
Writer
ZC3H13
p = 0.65
p = 0.058
p = 0.78
PD SD PR/CR
Writer
RBM15B
p = 0.51
p = 0.02
p = 0.55
PD SD PR/CR
F
Eraser
ALKBH5
p = 0.58
p = 0.13
p = 0.9
PD SD PR/CR
Eraser
FTO
p = 0.43
p = 0.004
p = 0.19
PD SD PR/CR

Supplementary Figure 2. The association of m6A regulator expression with the ICI response. (A-C) Box plot showing the expression of m6A readers (A), writers (B), and erasers (C) in the PD and CR groups using RNA-sequencing data from the Hugo2016_PD1_Melanoma cohort (n = 18 biologically independent samples). **(D-F)** Box plot showing the expression of m6A readers (D), writers (E), and erasers (F) in the PD, SD, and PR/CR groups using RNA-sequencing data from the Riaz2016_PD1_Melanoma cohort (n = 56 biologically independent samples). The upper, middle and lower horizontal lines of the box represent the upper, median and lower quartile respectively. Whiskers depict the smallest or largest values within 1.5-fold of the interquartile range, and the points outside the box represent outliers. Two-tailed unpaired Student's *t* test (A-C) and Kruskal-Wallis with Dunn's multiple comparison test (D-F) are reported. Source data are provided as a Source Data file.

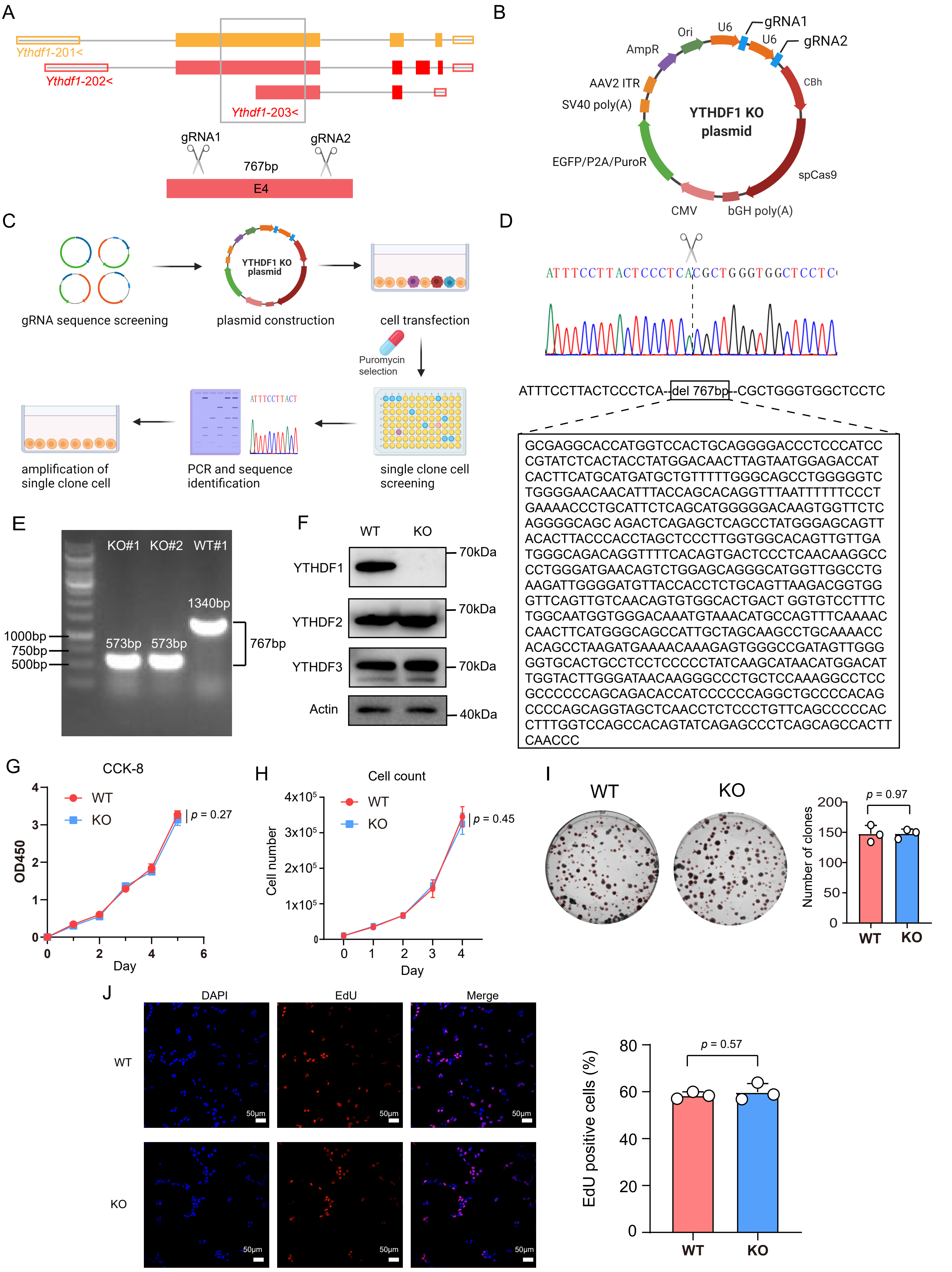
A
Ythdf1-201<
Ythdf1-202<
Ythdf1-203<
gRNA1
gRNA2
767bp
E4
B
YTHDF1 KO plasmid
gRNA1
gRNA2
U6
U6
Ori
AmpR
AAV2 ITR
SV40 poly(A)
EGFP/P2A/PuroR
CMV
bGH poly(A)
spCas9
CBh
C
gRNA sequence screening
plasmid construction
cell transfection
Puromycin selection
single clone cell screening
PCR and sequence identification
amplification of single clone cell
D
ATTTCCTTACTCCCTCA--del 767bp--CGCTGGGTGGCTCCTC
GCGAGGCACCATGGTCCACTGCAGGGGACCCTCCCATCC
CGTATCTCACTACCTATGGACAACTTAGTAATGGAGACCAT
CACTTCATGCATGATGCTGTTTTTGGGCAGCCTGGGGGTC
TGGGGAACAACATTTACCAGCACAGGTTTAATTTTTTCCCT
GAAAACCCTGCATTCTCAGCATGGGGGACAAGTGGTTCTC
AGGGGCAGC AGACTCAGAGCTCAGCCTATGGGAGCAGTT
ACACTTACCCACCTAGCTCCCTTGGTGGCACAGTTGTTGA
TGGGCAGACAGGTTTTCACAGTGACTCCCTCAACAAGGCC
CCTGGGATGAACAGTCTGGAGCAGGGCATGGTTGGCCTG
AAGATTGGGGATGTTACCACCTCTGCAGTTAAGACGGTGG
GTTCAGTTGTCAACAGTGTGGCACTGACT GGTGTCCTTTC
TGGCAATGGTGGGACAAATGTAAACATGCCAGTTTCAAAAC
CAACTTCATGGGCAGCCATTGCTAGCAAGCCTGCAAAACC
ACAGCCTAAGATGAAAACAAAGAGTGGGCCGATAGTTGGG
GGTGCACTGCCTCCTCCCCCTATCAAGCATAACATGGACAT
TGGTACTTGGGATAACAAGGGCCCTGCTCCAAAGGCCTCC
GCCCCCCAGCAGACACCATCCCCCCAGGCTGCCCCACAG
CCCCAGCAGGTAGCTCAACCTCTCCCTGTTCAGCCCCCAC
CTTTGGTCCAGCCACAGTATCAGAGCCCTCAGCAGCCACTT
CAACCC
E
KO#1
KO#2
WT#1
1340bp
573bp
573bp
767bp
1000bp
750bp
500bp
F
WT
KO
YTHDF1
YTHDF2
YTHDF3
Actin
70kDa
70kDa
70kDa
40kDa
G
CCK-8
WT
KO
p = 0.27
OD450
Day
H
Cell count
WT
KO
p = 0.45
Cell number
Day
I
WT
KO
p = 0.97
Number of clones
WT
KO
J
DAPI
EdU
Merge
WT
KO
50μm
p = 0.57
EdU positive cells (%)
WT
KO

Supplementary Figure 3. Establishment and validation of CRISPR/Cas9-mediated *Ythdf1* knockout cell clones. (A) Fragment knockout strategy for *Ythdf1* depletion. **(B)** Structure of the *Ythdf1* KO plasmid. **(C)** Workflow for the generation of *Ythdf1* knockout cell clones. WT cells were exposed to the same procedure as KO cells except for transfection with the scramble plasmid. **(D-F)** Validation of *Ythdf1* depletion by Sanger sequencing (D), PCR (E), and western blotting (F). One of two representative experiments with similar results is shown. **(G)** Cell viability (n = 3 biologically independent samples per group). Two-tailed unpaired Student's *t* test. Data are presented as mean values +/- SD. **(H)** Cell growth (n = 3 biologically independent samples per group). Two-tailed unpaired Student's *t* test. Data are presented as mean values +/- SD. **(I)** Clonogenic assay (n = 3 biologically independent samples per group). Two-tailed unpaired Student's *t* test. Data are presented as mean values +/- SD. **(J)** Visualization of DNA replication by EdU incorporation (n = 3 biologically independent samples per group). Blue = DAPI, and red = EdU. Scale bar, 50 μm. Two-tailed unpaired Student's *t* test. Data are presented as mean values +/- SD. P values were determined using a two-tailed Student's t test (NS $P > 0.05$). Source data are provided as a Source Data file.

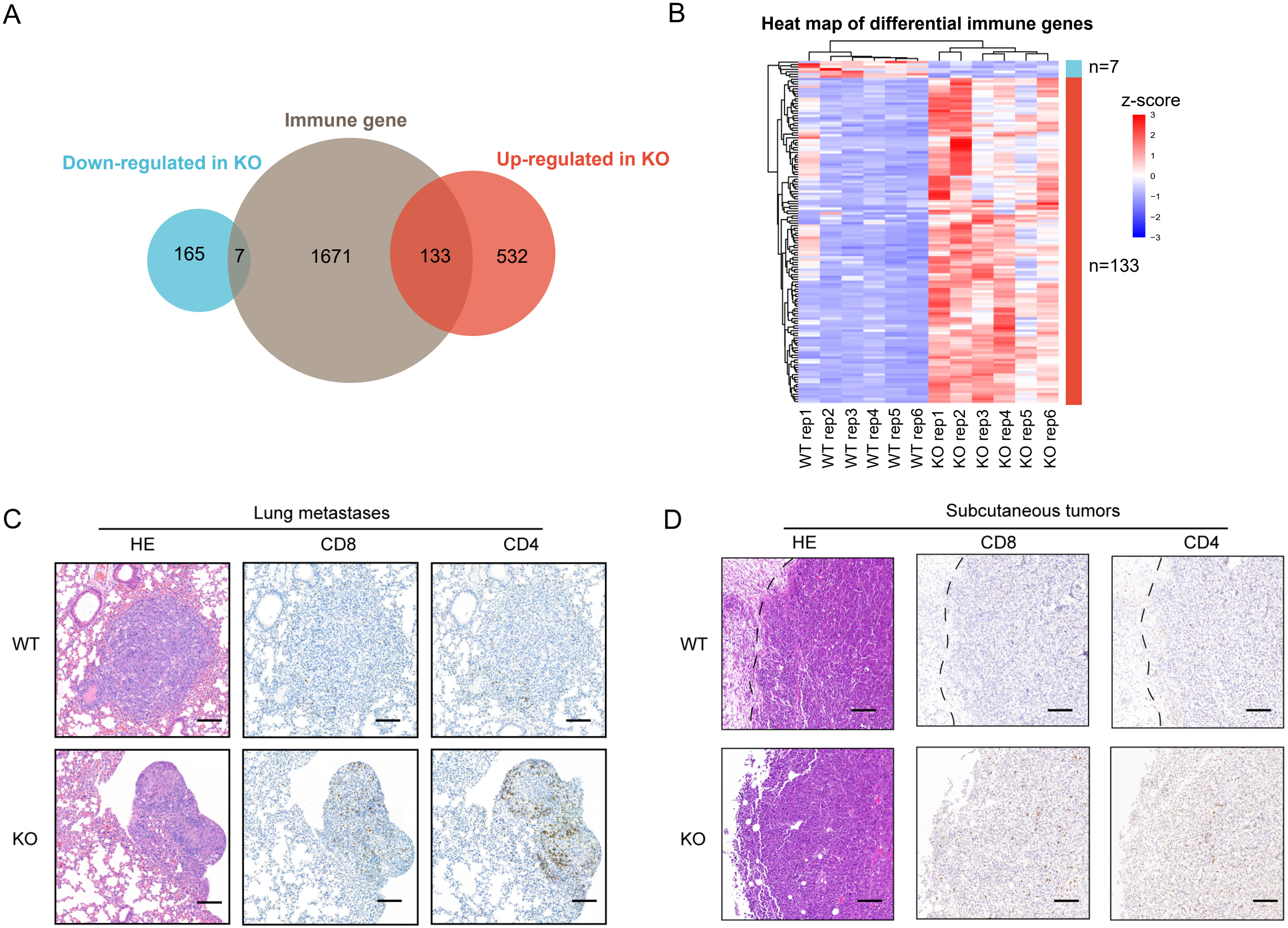
A
Immune gene
Down-regulated in KO
Up-regulated in KO
165
7
1671
133
532
B
Heat map of differential immune genes
n=7
n=133
z-score
3
2
1
0
−1
−2
−3
WT rep1
WT rep2
WT rep3
WT rep4
WT rep5
WT rep6
KO rep1
KO rep2
KO rep3
KO rep4
KO rep5
KO rep6
C
Lung metastases
HE
CD8
CD4
WT
KO
D
Subcutaneous tumors
HE
CD8
CD4
WT
KO

Supplementary Figure 4. Identification of differentially expressed immune-relevant genes and tumor-infiltrating T cells. (A) Venn diagram presenting the overlap of differentially expressed genes and immune-relevant genes. The immune-relevant gene list was obtained from the ImmPort database (https://www.immport.org/shared/genelists). **(B)** Heatmap of overlapping genes. Among the 140 overlapping immune-relevant genes, 133 were upregulated while 7 were downregulated in *Ythdf1*-KO tumors inoculated in C57BL/6J mice (n = 6 per group). **(C, D)** Immunohistochemical staining for $CD4^+$ and $CD8^+$ T cells in B16/F10 lung metastases (C) and subcutaneous tumors (D). Scale bar, 100 μm. One of two representative experiments with similar results is shown. Source data are provided as a Source Data file.

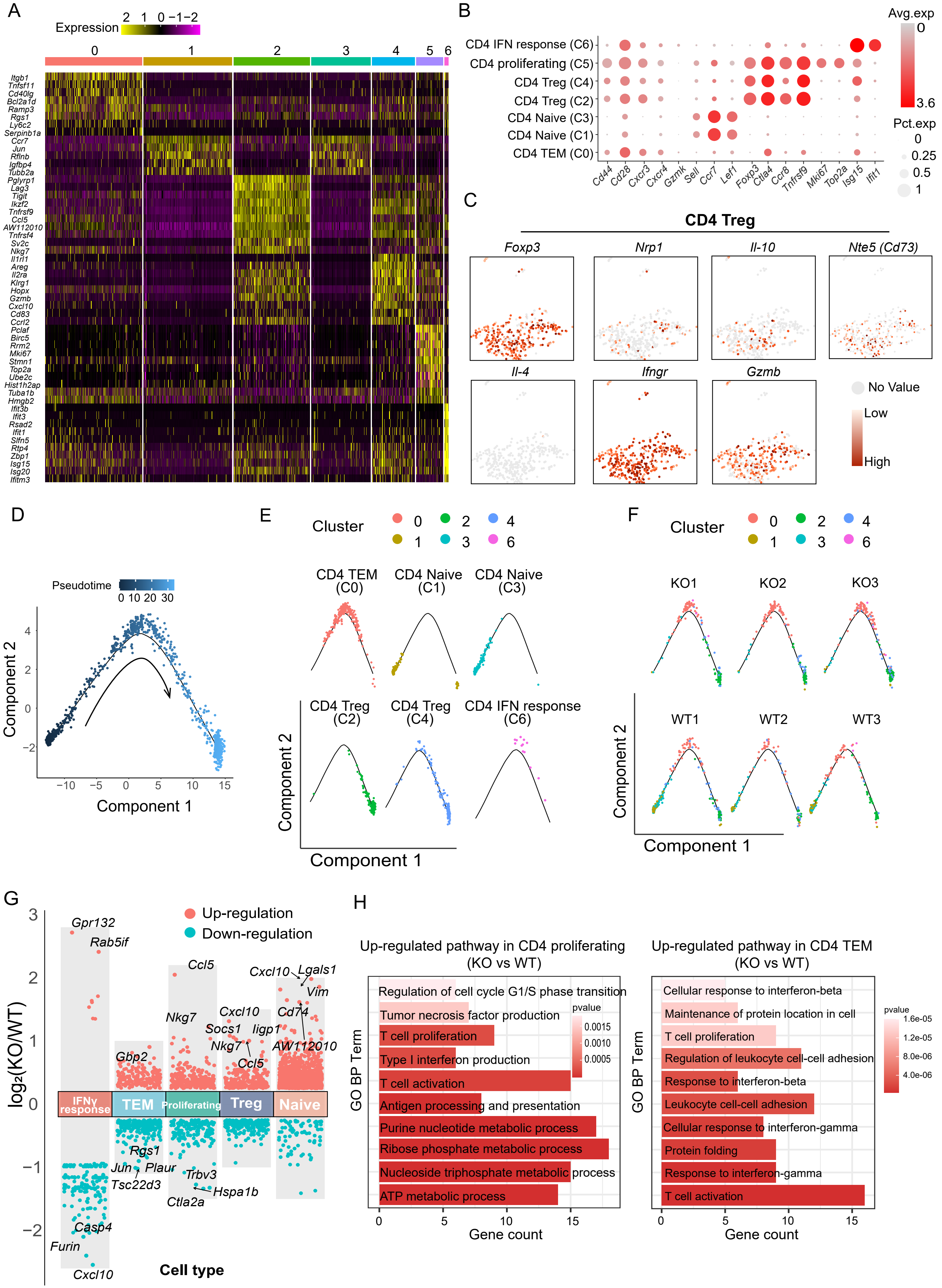

A
Expression
B
Avg.exp
Pct.exp
CD4 IFN response (C6)
CD4 proliferating (C5)
CD4 Treg (C4)
CD4 Treg (C2)
CD4 Naive (C3)
CD4 Naive (C1)
CD4 TEM (C0)
C
CD4 Treg
Foxp3
Nrp1
Il-10
Nte5 (Cd73)
Il-4
Ifngr
Gzmb
No Value
Low
High
D
Pseudotime
Component 2
Component 1
E
Cluster
CD4 TEM (C0)
CD4 Naive (C1)
CD4 Naive (C3)
CD4 Treg (C2)
CD4 Treg (C4)
CD4 IFN response (C6)
F
KO1
KO2
KO3
WT1
WT2
WT3
G
Up-regulation
Down-regulation
log2(KO/WT)
IFNγ response
TEM
Proliferating
Treg
Naive
Cell type
H
Up-regulated pathway in CD4 proliferating (KO vs WT)
GO BP Term
Regulation of cell cycle G1/S phase transition
Tumor necrosis factor production
T cell proliferation
Type I interferon production
T cell activation
Antigen processing and presentation
Purine nucleotide metabolic process
Ribose phosphate metabolic process
Nucleoside triphosphate metabolic process
ATP metabolic process
Gene count
pvalue
Up-regulated pathway in CD4 TEM (KO vs WT)
Cellular response to interferon-beta
Maintenance of protein location in cell
T cell proliferation
Regulation of leukocyte cell-cell adhesion
Response to interferon-beta
Leukocyte cell-cell adhesion
Cellular response to interferon-gamma
Protein folding
Response to interferon-gamma
T cell activation

Supplementary Figure 5. scRNA-seq analysis of $CD4^+$ T-cell subpopulations. (A) Heatmap of differentially expressed genes in each $CD4^+$ T-cell cluster. **(B)** Marker gene expression for each $CD4^+$ T-cell immune subpopulation, with dot color and size representing the averaged scaled expression (avg. exp. scale) value and percentage of marker gene expression (pct. exp), respectively. **(C)** t-SNE view of Tregs, color-coded by the indicated marker gene abundance. **(D)** Pseudotime analysis with dark blue indicating the starting point, the arrow denoting the trajectory across pseudotime and light blue representing the end of pseudotime. **(E, F)** Differentiation trajectories of cell clusters, color coded by cell cluster (E) and sample group (F). **(G)** Differential gene expression analysis showing up- and downregulated genes across all five $CD4^+$ T-cell subpopulations. **(H)** Gene ontology (GO) biological process (BP) enrichment for proliferating $CD4^+$ T cells and $CD4^+$ effector memory T cells (CD4 TEMs). Genes upregulated in the *Ythdf1*-KO group were collected for enrichment analysis. The one-tailed Fisher Exact test is reported. Source data are provided as a Source Data file.

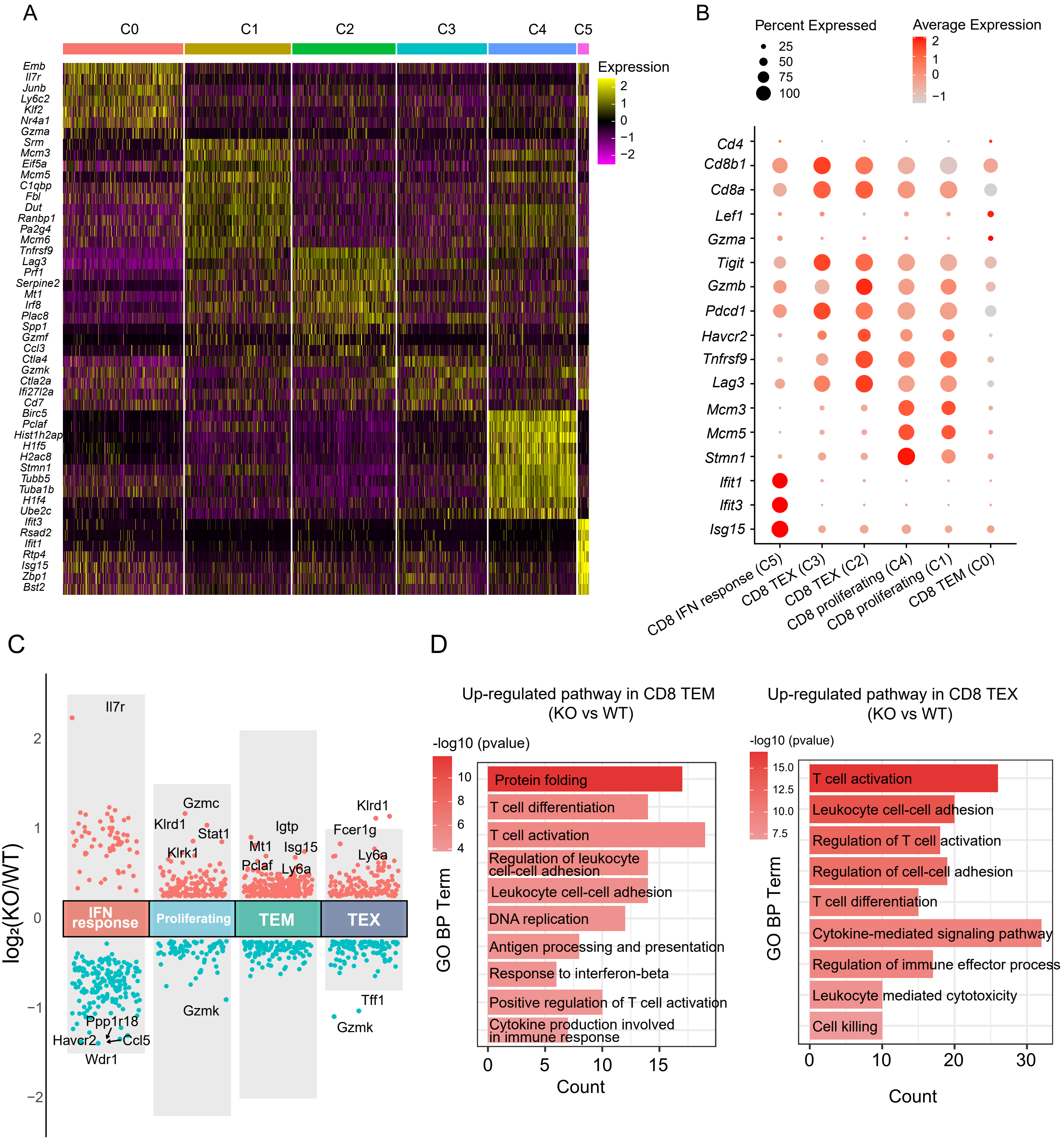

A
C0 C1 C2 C3 C4 C5
Expression
2 1 0 −1 −2
Emb
Il7r
Junb
Ly6c2
Klf2
Nr4a1
Gzma
Srm
Mcm3
Eif5a
Mcm5
C1qbp
Fbl
Dut
Ranbp1
Pa2g4
Mcm6
Tnfrsf9
Lag3
Prf1
Serpine2
Mt1
Irf8
Plac8
Spp1
Gzmf
Ccl3
Ctla4
Gzmk
Ctla2a
Ifi27l2a
Cd7
Birc5
Pclaf
Hist1h2ap
H1f5
H2ac8
Stmn1
Tubb5
Tuba1b
H1f4
Ube2c
Ifit3
Rsad2
Ifit1
Rtp4
Isg15
Zbp1
Bst2
B
Percent Expressed
25
50
75
100
Average Expression
2
1
0
−1
Cd4
Cd8b1
Cd8a
Lef1
Gzma
Tigit
Gzmb
Pdcd1
Havcr2
Tnfrsf9
Lag3
Mcm3
Mcm5
Stmn1
Ifit1
Ifit3
Isg15
CD8 IFN response (C5)
CD8 TEX (C3)
CD8 TEX (C2)
CD8 proliferating (C4)
CD8 proliferating (C1)
CD8 TEM (C0)
C
log₂(KO/WT)
2 1 0 −1 −2
Il7r
Ppp1r18
Havcr2
Ccl5
Wdr1
Gzmc
Klrd1
Stat1
Klrk1
Gzmk
Igtp
Mt1
Isg15
Pclaf
Ly6a
Klrd1
Fcer1g
Ly6a
Tff1
Gzmk
IFN response
Proliferating
TEM
TEX
D
Up-regulated pathway in CD8 TEM (KO vs WT)
-log10 (pvalue)
10 8 6 4
GO BP Term
Protein folding
T cell differentiation
T cell activation
Regulation of leukocyte cell-cell adhesion
Leukocyte cell-cell adhesion
DNA replication
Antigen processing and presentation
Response to interferon-beta
Positive regulation of T cell activation
Cytokine production involved in immune response
0 5 10 15
Count
Up-regulated pathway in CD8 TEX (KO vs WT)
-log10 (pvalue)
15.0 12.5 10.0 7.5
GO BP Term
T cell activation
Leukocyte cell-cell adhesion
Regulation of T cell activation
Regulation of cell-cell adhesion
T cell differentiation
Cytokine-mediated signaling pathway
Regulation of immune effector process
Leukocyte mediated cytotoxicity
Cell killing
0 10 20 30
Count

Supplementary Figure 6. scRNA-seq analysis of CD8$^+$ T-cell subpopulations. (A) Heatmap of differentially expressed genes in each CD8$^+$ T-cell cluster. **(B)** Marker gene expression for each CD8$^+$ T-cell immune subpopulation, with dot color and size representing the averaged scaled expression (avg. exp. scale) value and percentage of marker gene expression (pct. exp), respectively. **(C)** Differential gene expression analysis showing up- and down-regulated genes across all four CD8$^+$ T-cell subpopulations. **(D)** GO biological process enrichment for CD8$^+$ effector memory T cells (CD8$^+$ TEMs) and exhausted CD8$^+$ T cells (CD8$^+$ TEXs). Genes upregulated in the *Ythdf1*-KO group were collected for enrichment analysis. The one-tailed Fisher Exact test is reported. Source data are provided as a Source Data file.

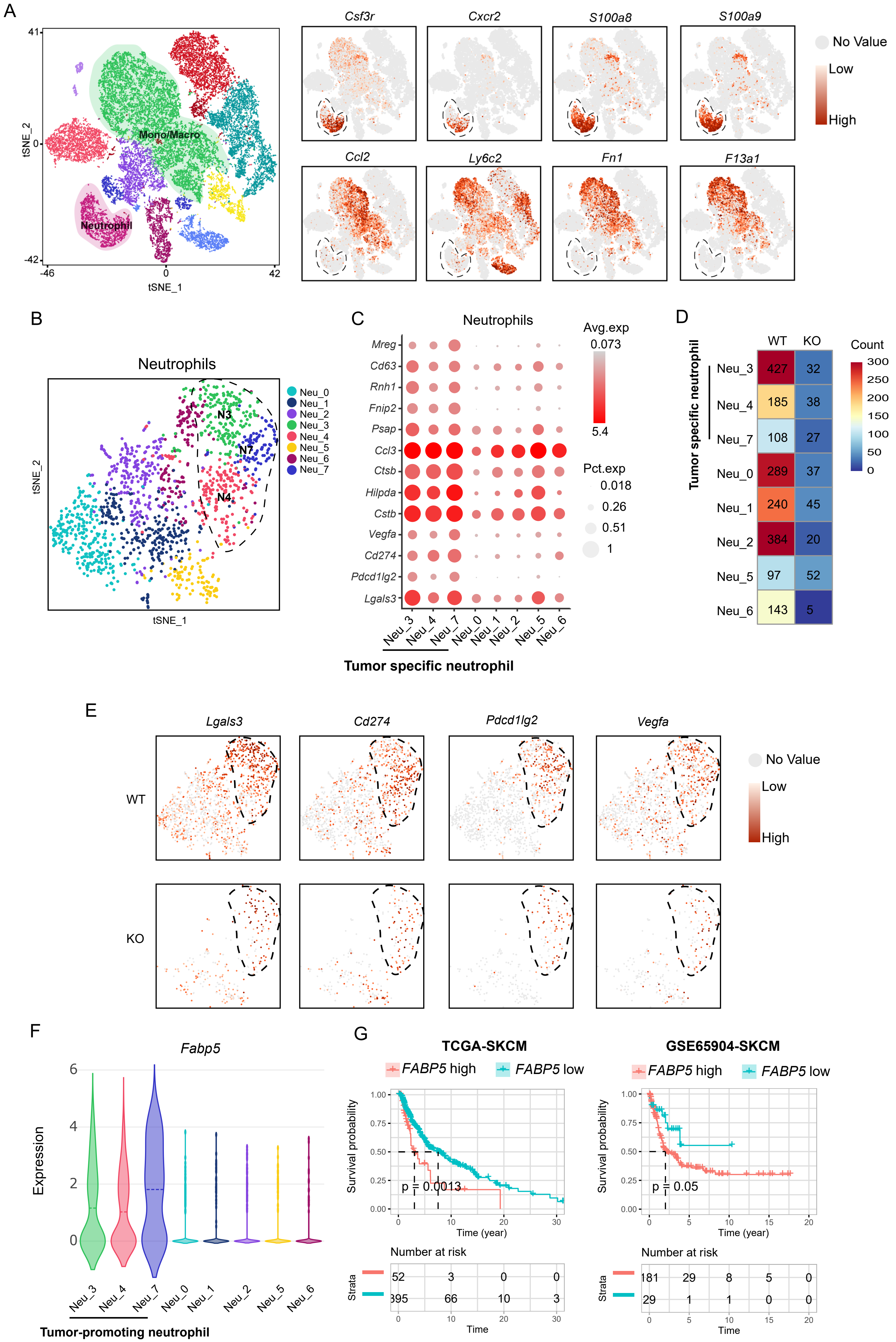

A
tSNE_2
tSNE_1
41
0
-42
-46
0
42
Mono/Macro
Neutrophil
Csf3r
Cxcr2
S100a8
S100a9
Ccl2
Ly6c2
Fn1
F13a1
No Value
Low
High
B
Neutrophils
N3
N7
N4
Neu_0
Neu_1
Neu_2
Neu_3
Neu_4
Neu_5
Neu_6
Neu_7
tSNE_2
tSNE_1
C
Neutrophils
Mreg
Cd63
Rnh1
Fnip2
Psap
Ccl3
Ctsb
Hilpda
Cstb
Vegfa
Cd274
Pdcd1lg2
Lgals3
Neu_3
Neu_4
Neu_7
Neu_0
Neu_1
Neu_2
Neu_5
Neu_6
Tumor specific neutrophil
Avg.exp
0.073
5.4
Pct.exp
0.018
0.26
0.51
1
D
Tumor specific neutrophil
WT
KO
Neu_3 427 32
Neu_4 185 38
Neu_7 108 27
Neu_0 289 37
Neu_1 240 45
Neu_2 384 20
Neu_5 97 52
Neu_6 143 5
Count
300
250
200
150
100
50
0
E
Lgals3
Cd274
Pdcd1lg2
Vegfa
WT
KO
No Value
Low
High
F
Fabp5
Expression
6
4
2
0
Neu_3
Neu_4
Neu_7
Neu_0
Neu_1
Neu_2
Neu_5
Neu_6
Tumor-promoting neutrophil
G
TCGA-SKCM
FABP5 high
FABP5 low
Survival probability
1.00
0.75
0.50
0.25
0.00
p = 0.0013
0
10
20
30
Time (year)
Number at risk
Strata
52 3 0 0
395 66 10 3
Time
GSE65904-SKCM
FABP5 high
FABP5 low
Survival probability
p = 0.05
0
5
10
15
20
Time (year)
Number at risk
Strata
181 29 8 5 0
29 1 1 0 0
Time

Supplementary Figure 7. scRNA-seq analysis of neutrophil subpopulations. (A) t-SNE view of neutrophil markers, color coded by the indicated marker gene abundance. **(B)** t-SNE plot of neutrophil subpopulations, color coded by cell cluster. **(C)** Marker gene expression for each neutrophil subpopulation, with dot color and size representing the averaged scaled expression (avg. exp. scale) value and percentage of marker gene expression (pct. exp), respectively. **(D)** Heatmap of the cell count for each neutrophil subpopulation. **(E)** t-SNE view of neutrophils, color coded by the tumor-promoting genes abundance. **(F)** Violin plot of *Fabp5* expression for each neutrophil subpopulation. **(G)** Kaplan–Meier OS curve for melanoma patients with high or low FABP5 expression using RNA-sequencing data from the TCGA and GEO databases. Two-sided log-rank test.
Source data are provided as a Source Data file.

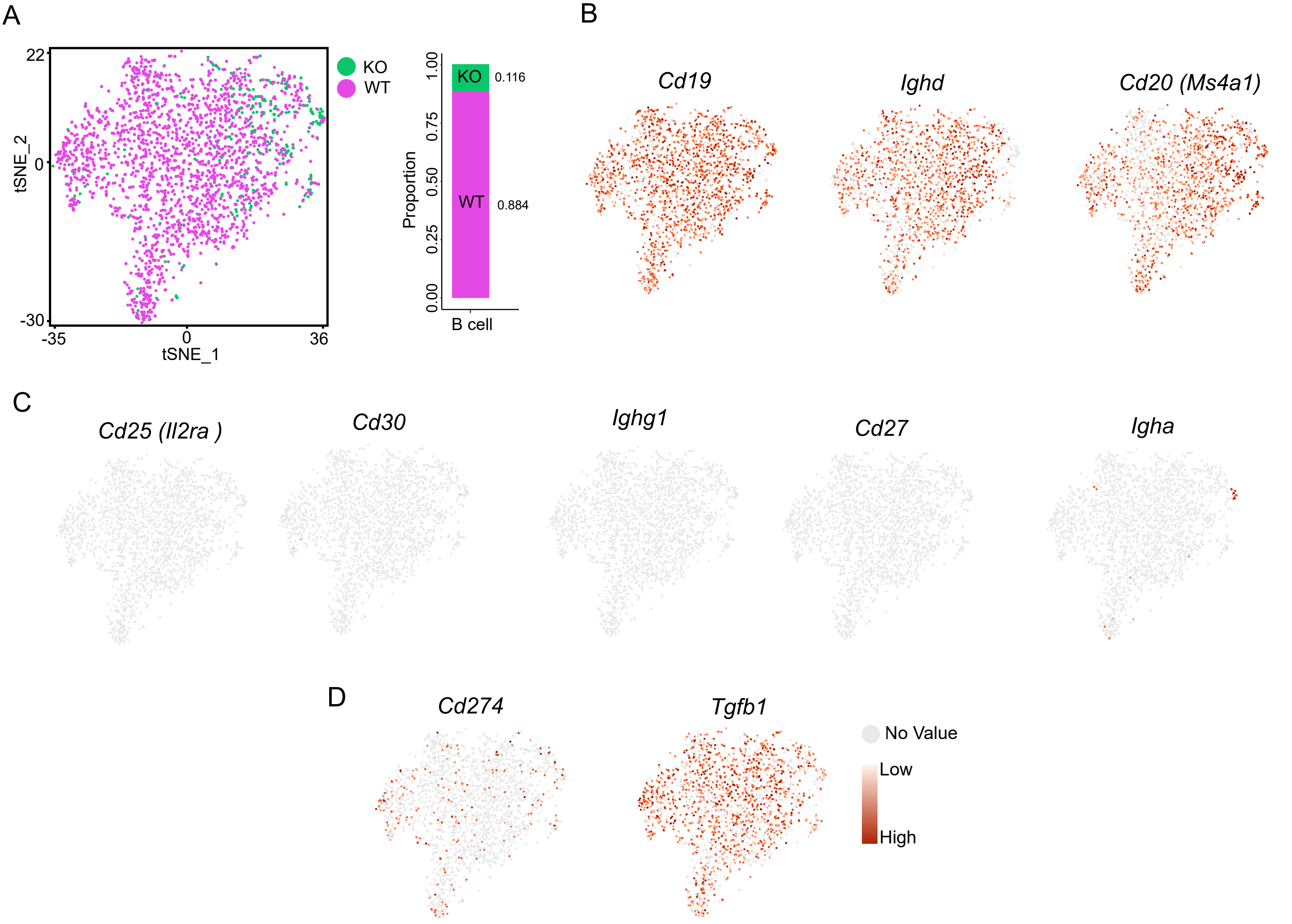

A
22
0
-30
-35
0
36
tSNE_2
tSNE_1
KO
WT
1.00
0.75
0.50
0.25
0.00
Proportion
KO
0.116
WT
0.884
B cell
B
Cd19
Ighd
Cd20 (Ms4a1)
C
Cd25 (Il2ra)
Cd30
Ighg1
Cd27
Igha
D
Cd274
Tgfb1
No Value
Low
High

Supplementary Figure 8. scRNA-seq analysis of B cells. (A) t-SNE plot of B-cells subpopulations, color coded by sample group, and bar plot depicting the ratios of B cells in WT and *Ythdf1*-KO tumors. **(B)** t-SNE view of naive B-cell markers (*Cd19*, *Ighd*, and *Cd20*), color coded by the indicated marker gene abundance. **(C)** t-SNE view of activated B-cell markers (*Cd25* and *Cd30*), plasma cell markers (*Ighg1* and *Cd27*), or memory B-cell markers (*Cd27*, *Igha*, and *Ighg1*), color coded by the indicated marker gene abundance. **(D)** t-SNE plot of immunosuppressive molecules (*Cd274* and *Tgfb1*), color coded by the indicated marker gene abundance. Source data are provided as a Source Data file.

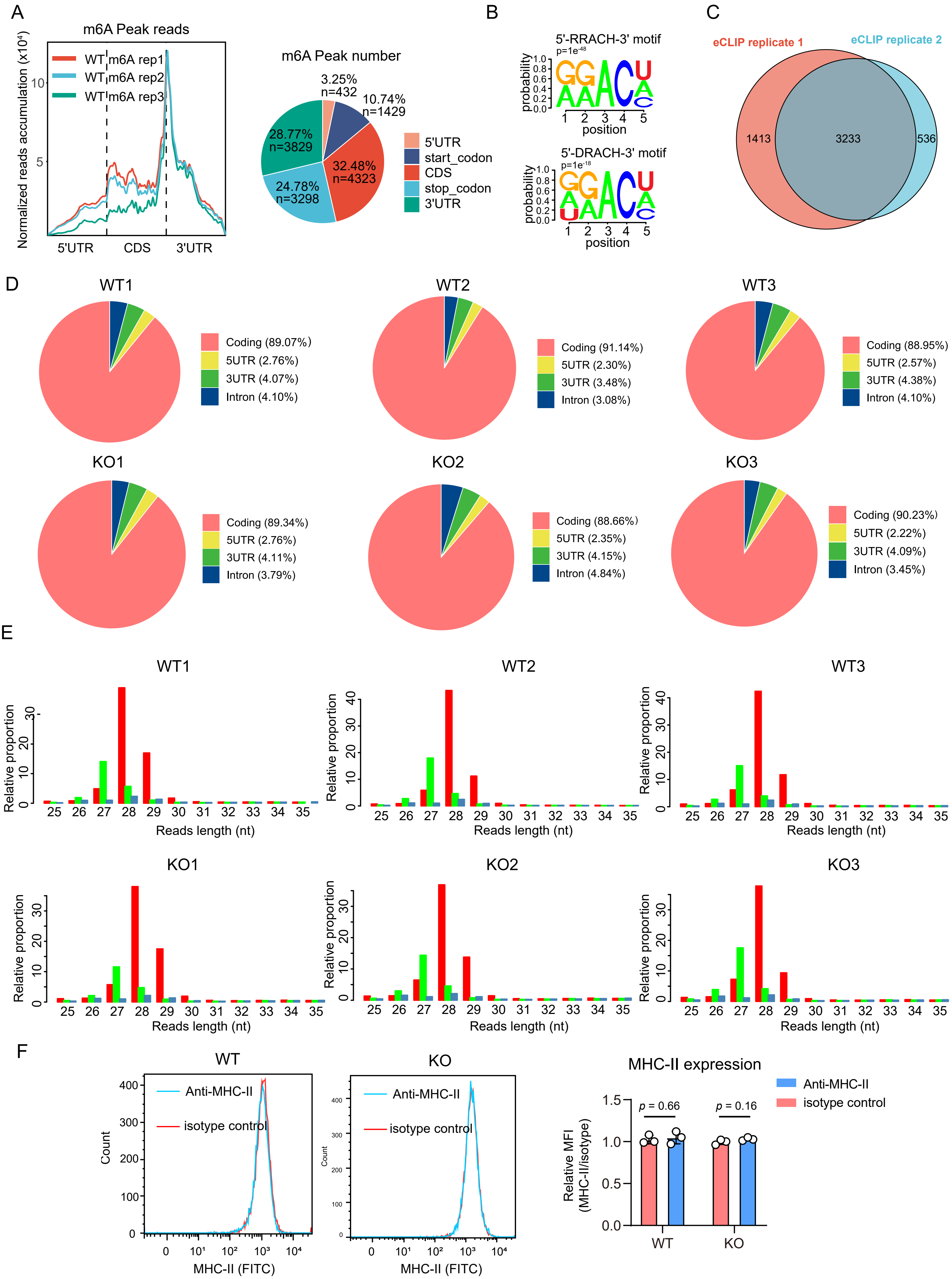
A
m6A Peak reads
WT m6A rep1
WT m6A rep2
WT m6A rep3
Normalized reads accumulation (x10⁴)
5'UTR
CDS
3'UTR
m6A Peak number
3.25% n=432
10.74% n=1429
28.77% n=3829
32.48% n=4323
24.78% n=3298
5'UTR
start_codon
CDS
stop_codon
3'UTR
B
5'-RRACH-3' motif
p=1e-48
probability
position
5'-DRACH-3' motif
p=1e-18
C
eCLIP replicate 1
eCLIP replicate 2
1413
3233
536
D
WT1
Coding (89.07%)
5UTR (2.76%)
3UTR (4.07%)
Intron (4.10%)
WT2
Coding (91.14%)
5UTR (2.30%)
3UTR (3.48%)
Intron (3.08%)
WT3
Coding (88.95%)
5UTR (2.57%)
3UTR (4.38%)
Intron (4.10%)
KO1
Coding (89.34%)
5UTR (2.76%)
3UTR (4.11%)
Intron (3.79%)
KO2
Coding (88.66%)
5UTR (2.35%)
3UTR (4.15%)
Intron (4.84%)
KO3
Coding (90.23%)
5UTR (2.22%)
3UTR (4.09%)
Intron (3.45%)
E
WT1
WT2
WT3
KO1
KO2
KO3
Relative proportion
Reads length (nt)
F
WT
KO
Anti-MHC-II
isotype control
Count
MHC-II (FITC)
MHC-II expression
Relative MFI (MHC-II/isotype)
p = 0.66
p = 0.16
WT
KO

Supplementary Figure 9. m6A MeRIP-seq, eCLIP-seq, and ribo-seq analysis. (A) The distribution of m6A peak reads and proportions in the 5'UTR, start codon, CDS, stop codon and 3'UTR in mRNA transcripts. **(B)** The m6A motif detected by MEME motif analysis. The RRACH and DRACH (D=G/A/U, R=G/A, H=A/U/C) conserved sequence motifs for m6A-containing peak regions. **(C)** Overlap of YTHDF1-binding transcripts revealed from eCLIP-seq of two biological replicates. **(D)** The distribution and percentage of ribosome-protected transcripts in the coding, 5'UTR, 3'UTR, and intronic regions. **(E)** Bar plot illustrating the count of each ribosome-protected fragment, with colors representing different frames. **(F)** Quantitative estimates of cell surface MHC-II levels in WT and *Ythdf1*-KO B16F10 cells (n = 3 biologically independent samples per group). Two-tailed unpaired Student's *t* test. Data are presented as mean values +/- SD. Source data are provided as a Source Data file.

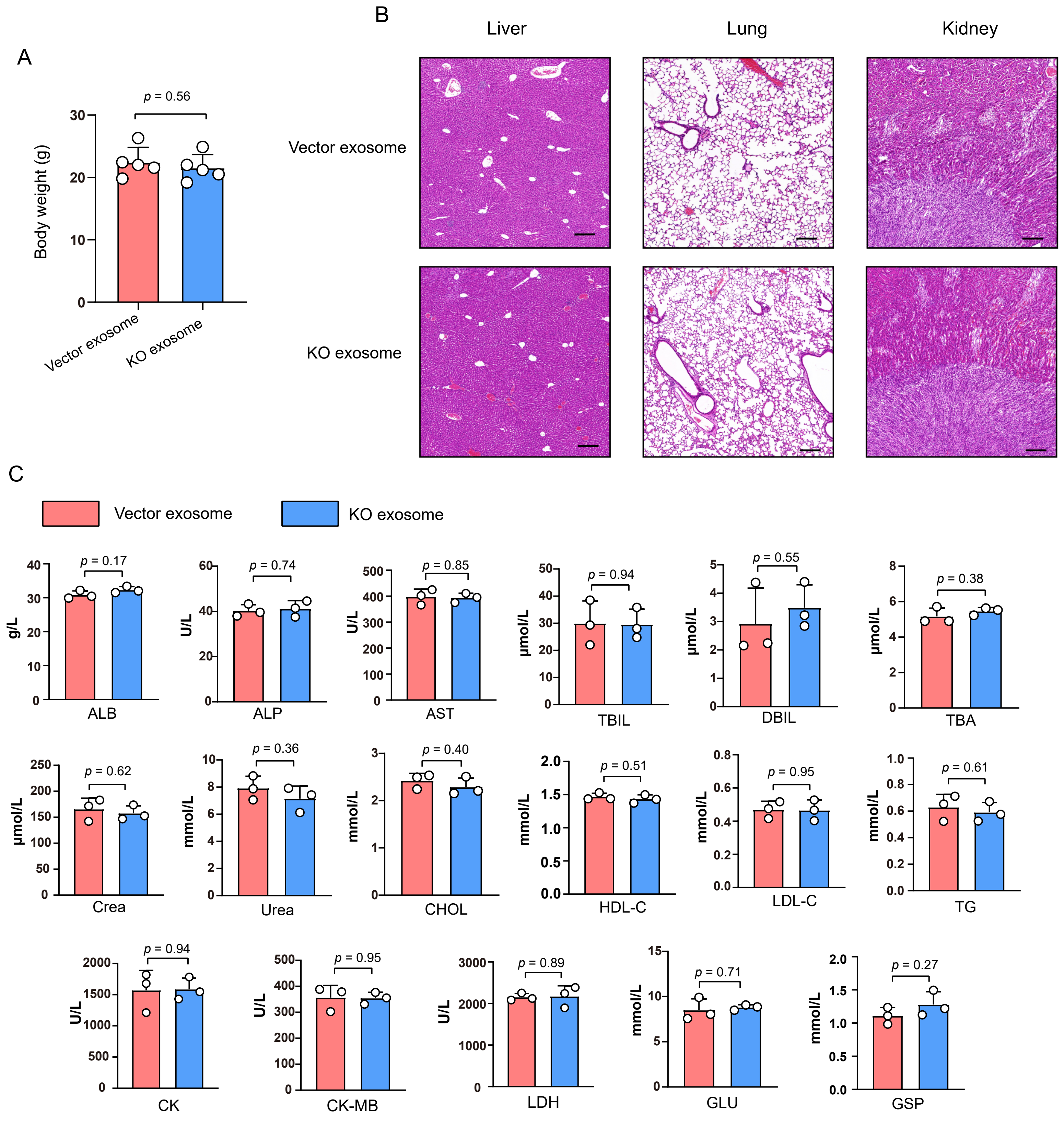

A
B
C
p = 0.56
Body weight (g)
Vector exosome
KO exosome
Liver
Lung
Kidney
Vector exosome
KO exosome
Vector exosome
KO exosome
p = 0.17
ALB
g/L
p = 0.74
ALP
U/L
p = 0.85
AST
U/L
p = 0.94
TBIL
μmol/L
p = 0.55
DBIL
μmol/L
p = 0.38
TBA
μmol/L
p = 0.62
Crea
μmol/L
p = 0.36
Urea
mmol/L
p = 0.40
CHOL
mmol/L
p = 0.51
HDL-C
mmol/L
p = 0.95
LDL-C
mmol/L
p = 0.61
TG
mmol/L
p = 0.94
CK
U/L
p = 0.95
CK-MB
U/L
p = 0.89
LDH
U/L
p = 0.71
GLU
mmol/L
p = 0.27
GSP
mmol/L

Supplementary Figure 10. Toxicity test of engineered exosomes treatment. (A) Body weight (n = 5 biologically independent samples per group). Two-tailed unpaired Student's *t* test. Data are presented as mean values +/- SD. **(B)** HE staining of liver, lung, and kidney tissue. Scale bar, 200 μm. One of three representative experiments with similar results is shown. **(C)** Evaluation of blood biochemistry, including albumin (ALB), alkaline phosphatase (ALP), aspartate aminotransferase (AST), total bilirubin (TBIL), direct bilirubin (DBIL), total bile acid (TBA), creatinine (Crea), Urea, cholesterol (CHOL), high density lipoprotein cholesterol (HDL-C), low density lipoprotein cholesterol (LDL-C), triglyceride (TG), creatine kinase (CK), Creatine kinase MB isoenzyme (CK-MB), lactic dehydrogenase (LDH), glucose (GLU), and glycated serum protein (GSP). n = 3 biologically independent samples per group.Two-tailed unpaired Student's *t* test. Data are presented as mean values +/- SD. Source data are provided as a Source Data file.

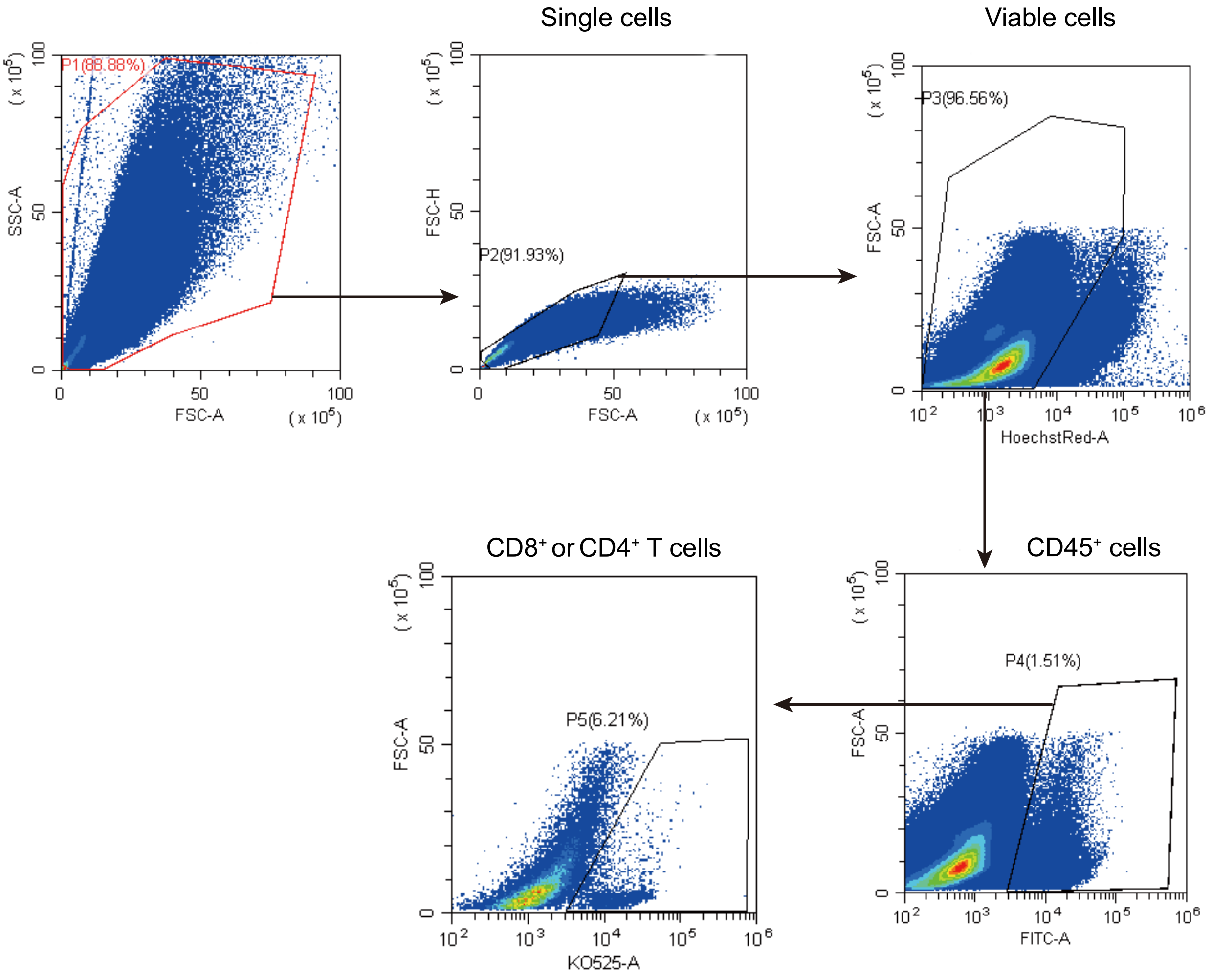

Supplementary Figure 11. Gating strategy of tumor-infiltrating $CD4^{+}$ and $CD8^{+}$ T cells.

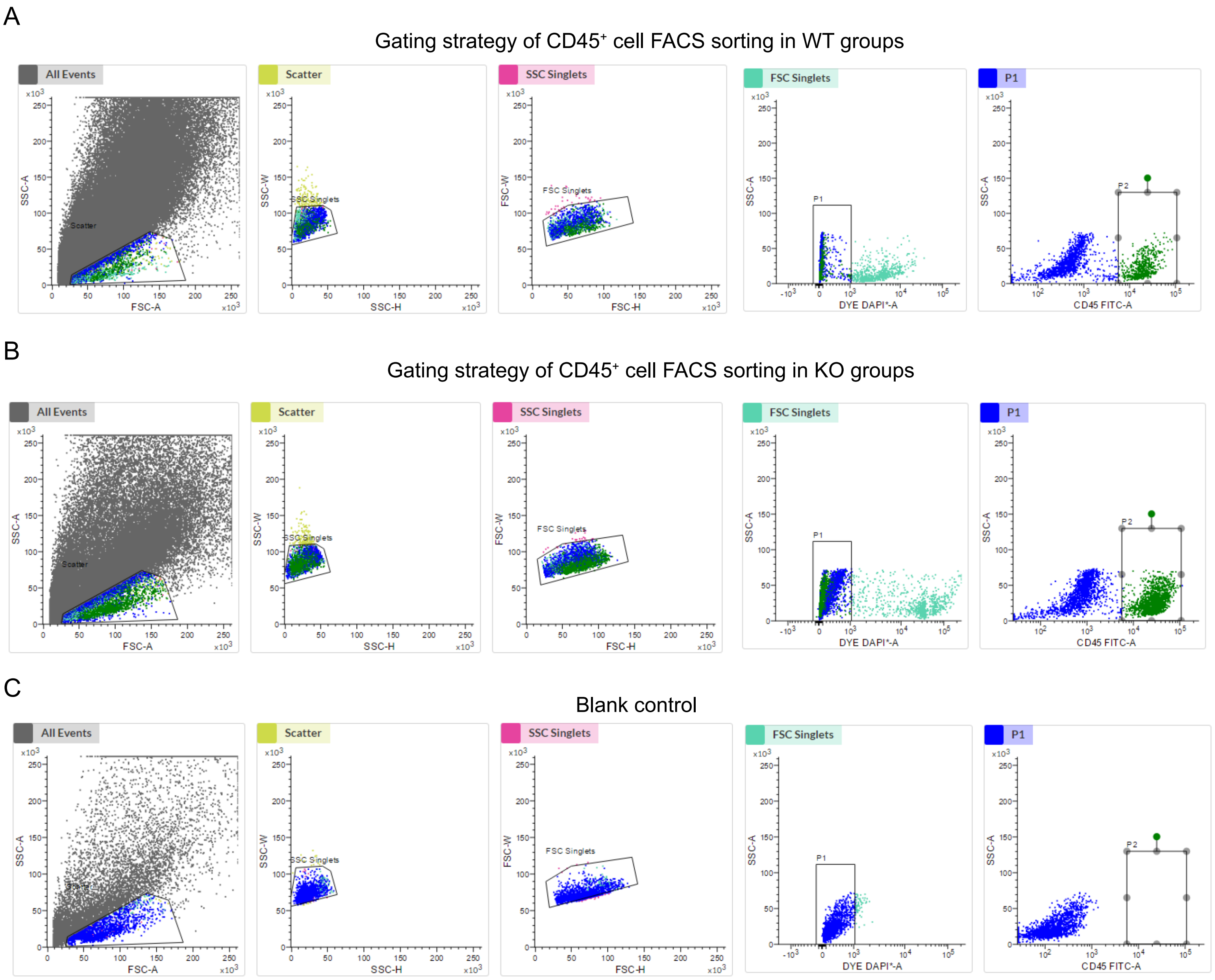

A
Gating strategy of CD45+ cell FACS sorting in WT groups
B
Gating strategy of CD45+ cell FACS sorting in KO groups
C
Blank control
All Events
Scatter
SSC Singlets
FSC Singlets
P1
P2
SSC-A
FSC-A
SSC-W
SSC-H
FSC-W
FSC-H
DYE DAPI*-A
CD45 FITC-A

Supplementary Figure 12. Flow-sorting of $CD45^{+}$ cells from tumor tissue. (A-C) Gating strategy of flow-sorting in WT, KO and blank control groups.
