# Supplementary material for: Tumor-intrinsic YTHDF1 drives immune evasion and resistance to immune checkpoint inhibitors via promoting MHC-I degradation
Source: Nat Commun. 2023 Jan 17;14:265. doi: 10.1038/s41467-022-35710-7 (PMC9845301; doi:10.1038/s41467-022-35710-7)

Immunoblot images depicted in Figure 1J

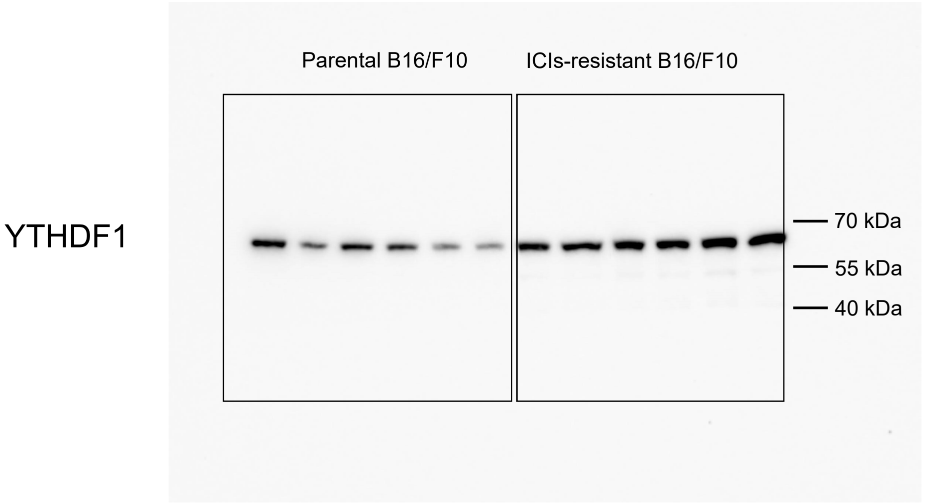

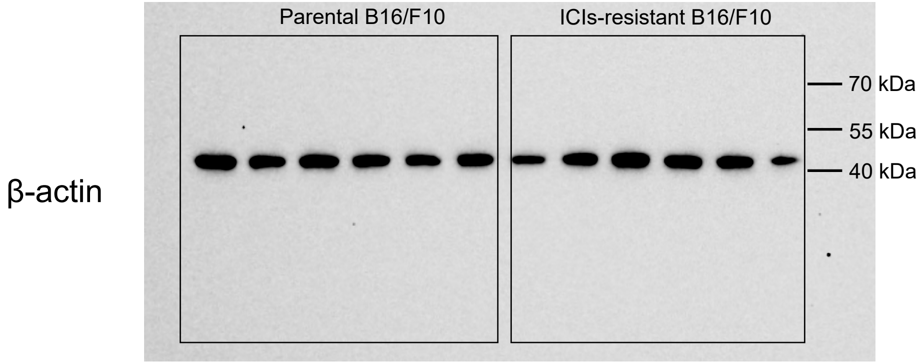

Immunoblot images depicted in Figure 8C

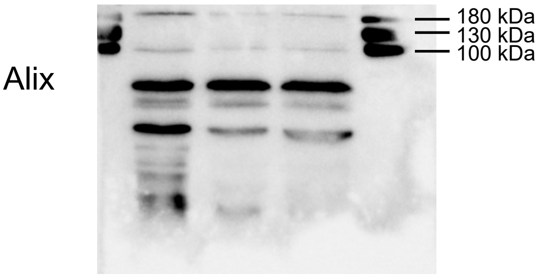

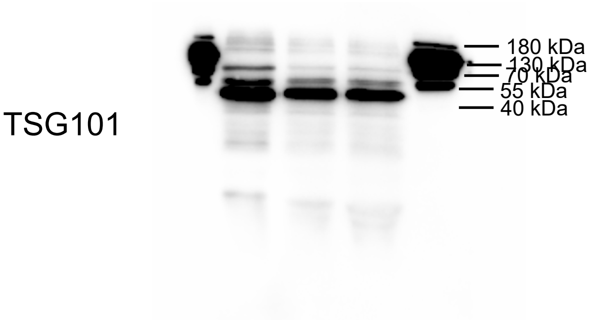

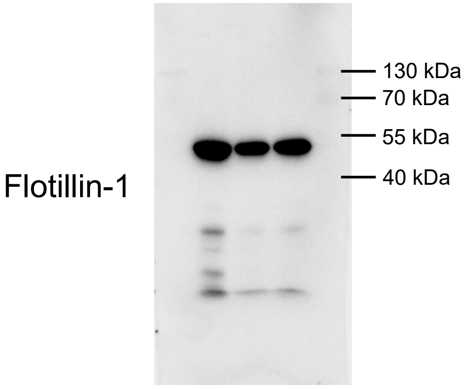

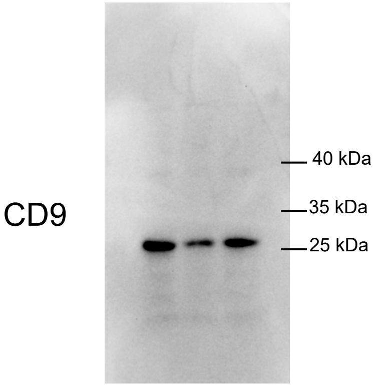

Immunoblot images depicted in Figure 8I

YTHDF1

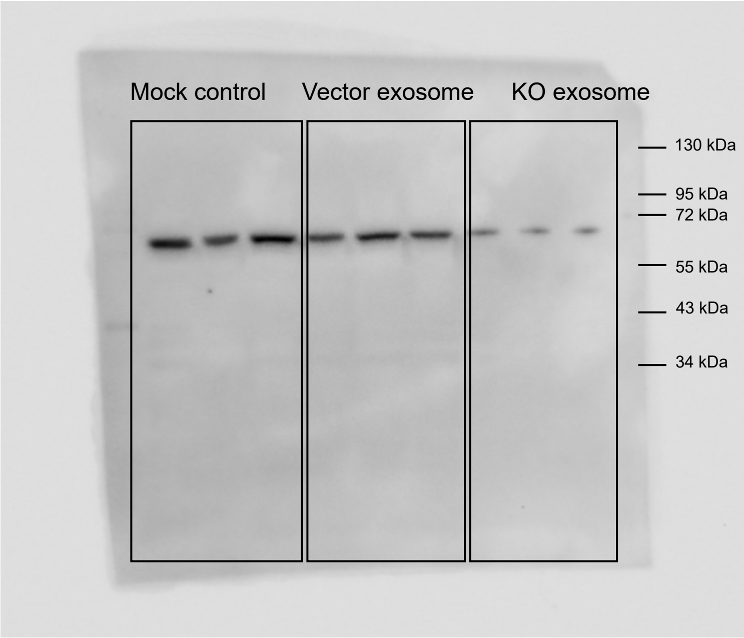

β-actin

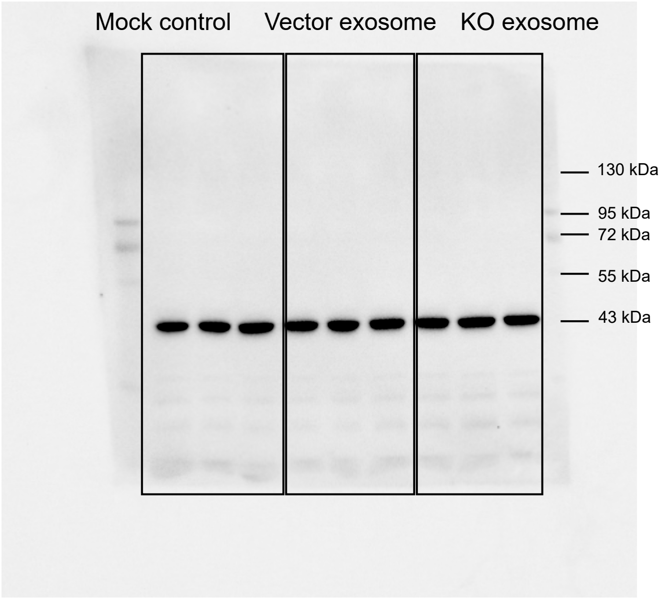

Immunoblot images depicted in supplementary Figure 3F

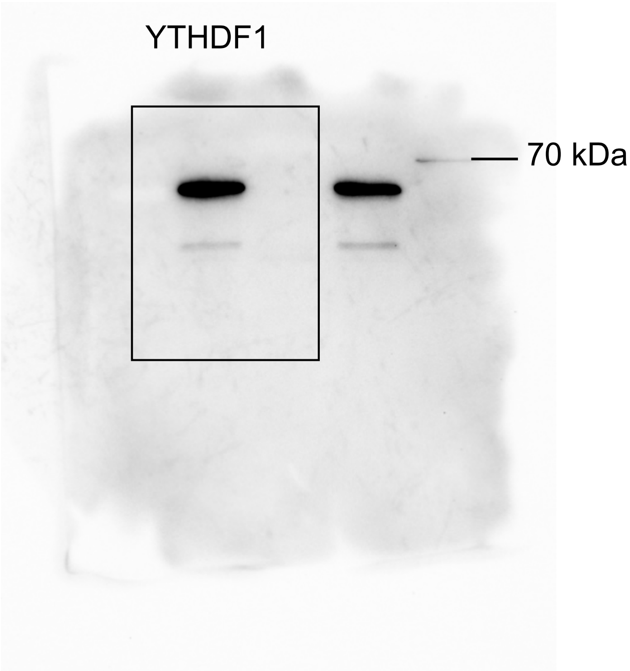

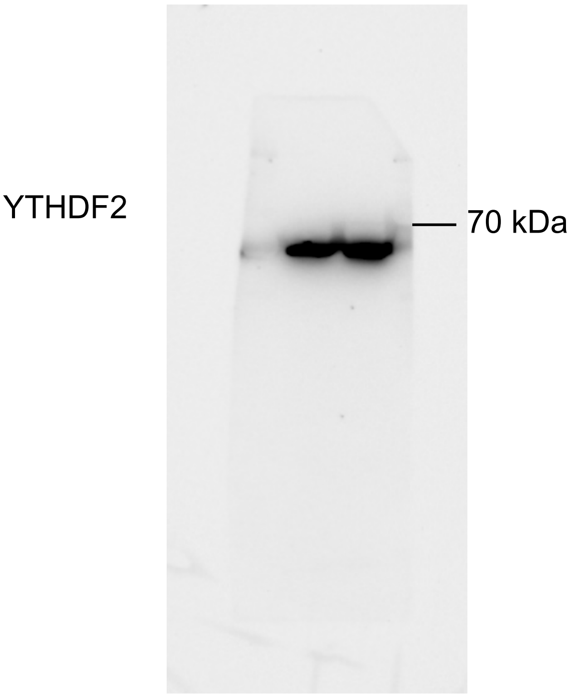

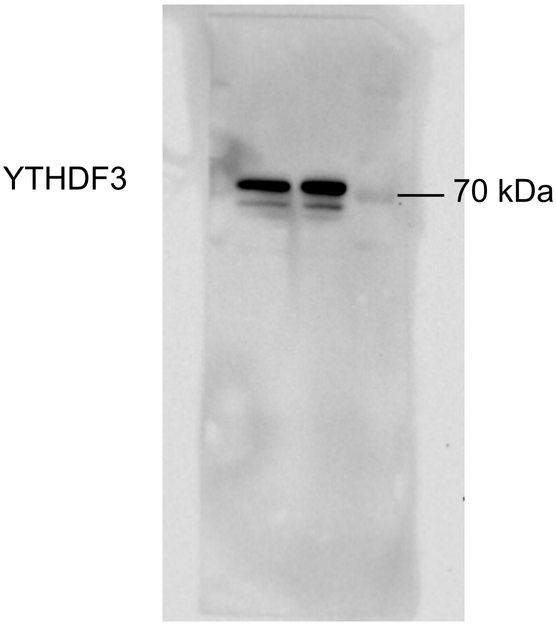

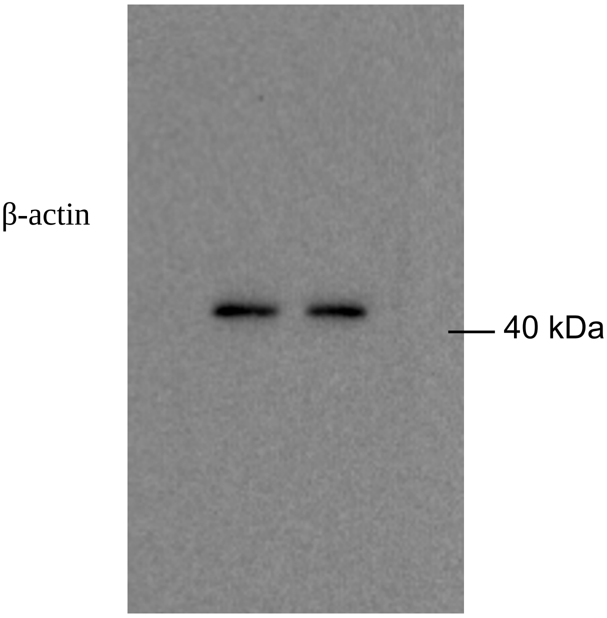

Supplement: Supplementary file 4 — Source Data [file 41467_2022_35710_MOESM4_ESM.zip › Source Data file/Uncropped gel images.pdf]
